# Reply: Accuracy of Smartwatches in the Detection of Atrial Fibrillation

**DOI:** 10.1016/j.jacadv.2026.102711

**Published:** 2026-04-20

**Authors:** Nelson I. Barrera, Yomary Jimenez, Guilherme Dagostin Carvalho

**Affiliations:** a SBH Health System, Bronx, New York, USA; b University of Florida, Gainesville, Florida, USA; c Dante Pazzanese Institute of Cardiology, Department of Cardiac Arrhythmias and Electrophysiology, São Paulo, Brazil

We thank Dr Beyls and colleagues and Dr Doggart for their thoughtful commentary on our study.^1^

Dr Doggart appropriately identifies analytical heterogeneity affecting device comparisons in our Central Illustration. Our Fitbit data originated from a single study by Mannhart et al,^2^ which employed intention-to-diagnose analysis (inconclusive results were counted as incorrect), whereas most other devices derived from multiple studies, excluding inconclusive results. This methodological heterogeneity compromises fair device-by-device comparisons. We have removed the Fitbit data and we thank Dr Doggart for this important observation.

We appreciate Dr Beyls and colleagues’ distinction between detection and diagnosis. While photoplethysmography and electrocardiogram (ECG) technologies differ mechanistically, our meta-regression found no significant difference in diagnostic accuracy when both were validated against gold standard references. However, we fully agree that clinical practice requires ECG confirmation of any smartwatch alert before clinical action, as current guidelines mandate.^3^ Algorithmic heterogeneity across devices is an important consideration. This heterogeneity reflects fundamental differences in device technologies. Proprietary algorithms, varying software updates, and diverse validation populations all contribute to device-specific performance, demonstrating that smartwatch accuracy is not uniform across manufacturers.

The studies included in our analysis were conducted under controlled conditions that varied substantially from everyday use. Noninterpretable recordings (10% to 15% in practice), which were often excluded from the studies we analyzed, represent a critical implementation barrier often excluded from studies. When included, diagnostic accuracy decreases substantially.^3^ Additionally, our 11% atrial fibrillation prevalence in our pooled analysis far exceeds what most clinicians encounter in general practice. In lower-prevalence settings, false positive alerts increase substantially, creating patient anxiety and unnecessary workload.

As the field of medicine and cardiology evolves, new technologies like smartwatches should be integrated thoughtfully as adjuncts, not competitors, to clinical ECG or medical confirmation. Their alerts can empower patients by prompting timely medical evaluation, but we reiterate that confirmation with standard diagnostic tools remains essential before any clinical action. We also agree that a critical gap in the literature persists regarding the performance of smartwatches in controlled environments vs real-world scenarios.

Nonetheless, alerts play a meaningful role in guiding patients to seek appropriate medical confirmation, and we hope our study serves as a foundation for future research aimed at evaluating real-world accuracy in clinical trials.
